# Sustained response to rituximab in an ANCA‐negative necrotizing vasculitis with initial detection of *Corynebacterium kroppenstedtii*

**DOI:** 10.1002/cti2.70119

**Published:** 2026-08-11

**Authors:** Nadine Al‐Azem, Anna Knothe, Jan‐Dirk Raguse, Inga Nierkamp, Achim Georg Beule, Claudia Rudack, Friedericke Müller, Anne Kerlikowki, Eva Wardelmann, Hermann Pavenstädt, Martin A Kriegel, Rebecca Hasseli

**Affiliations:** ^1^ Section of Rheumatology and Clinical Immunology, Department of Medicine D University Hospital Münster Münster Germany; ^2^ Department of Oral and Maxillofacial Surgery Fachklinik Hornheide Münster Germany; ^3^ Clinic for Ear, Nose, and Throat Disorders University Hospital Münster Münster Germany; ^4^ Center for Diseases of the Upper Airway University Clinic Münster Münster Germany; ^5^ Department of Otorhinolaryngology, Head and Neck Surgery University Medicine Greifswald Greifswald Germany; ^6^ Gerhard‐Domagk‐Institute of Pathology University Hospital Münster Münster Germany; ^7^ Department of Translational Rheumatology and Immunology, Institute for Musculoskelettal Medicine University of Münster Münster Germany; ^8^ Institute of Immunology University of Münster Münster Germany

**Keywords:** B cell depletion, infectious disease, inflammatory disease, orbital destruction

## Abstract

**Objectives:**

In this case, we present a patient with rapidly progressive unilateral destructive orbital and midface inflammation. Initial detection of *Corynebacterium kroppenstedtii* was followed by a consistent course of localised necrotizing small‐vessel vasculitis, which was responsive to B‐cell depletion.

**Methods:**

A 79‐year‐old woman presented with acute left periorbital pain, tearing and purulent discharge. The workup included serial microbiological cultures, fungal and mycobacterial testing, molecular diagnostics, CT/MRI/PET‐CT imaging, multiple surgical biopsies with histopathology and special stains, ANCA testing, and multidisciplinary treatments (antimicrobials, glucocorticoids, methotrexate and rituximab). The clinical course, laboratory work, imaging studies, histological analysis and response to treatment were thoroughly reviewed.

**Results:**

Initial conjunctival culture revealed *C*. *kroppenstedtii* and clindamycin and vancomycin was initiated. Despite escalating antimicrobials, the patient developed progressive inflammation with sinus destruction, cutaneous fistula and cheek abscess, requiring orbital exenteration. The biopsies revealed chronic inflammation with multinucleated giant cells, focal necrosis and small‐vessel vasculitis. ANCA remained negative, and subsequent testing was negative. High‐dose prednisolone improved the patient, prompting the discontinuation of methotrexate because of pancytopenia. Rituximab‐induced remission allowed for steroid tapering and rituximab maintenance, which controlled the disease without complications.

**Conclusion:**

This case illustrates a rare, localised ANCA‐negative necrotizing small‐vessel vasculitis with destructive orbital involvement and initial detection of *C. kroppenstedtii*. Although the pathogenic significance of this finding remains uncertain, the case highlights the importance of comprehensive microbiological and immunological evaluation in atypical destructive inflammatory disease. Sustained disease control was achieved with immunosuppressive therapy including rituximab after persistent infection had been excluded.

## Case report

*Corynebacterium kroppenstedtii* is a lipophilic, gram‐positive bacillus strongly associated with granulomatous lobular mastitis (GLM), a chronic inflammatory breast disease predominantly affecting women of reproductive age. The organism lacks mycolic acids and depends on lipid‐rich environments, which likely explains its marked tropism for breast tissue and its frequent identification as the principal pathogen in GLM.^1^ Histopathologically, *C. kroppenstedtii*‐associated GLM is characterised by lobulocentric granulomatous inflammation, often with xanthogranulomatous features and gram‐positive bacilli localised within lipid vacuoles.^1^ Once the organism has been identified, targeted treatment with lipophilic antibiotics should be considered, in conjunction with surgical intervention where clinically indicated.^1^

Small‐vessel vasculitis (SVV) comprises heterogeneous immune‐mediated disorders of venules, capillaries and arterioles, caused by autoimmune mechanisms, drugs or infections. Microbiome studies in vasculitis show altered skin and blood microbial profiles, including increased Corynebacteriales, but no direct association with *C. kroppenstedtii* has been demonstrated.^2^ Infectious triggers of SVV are primarily hepatitis B and C viruses and *Staphylococcus aureus*, rather than non‐diphtherial *Corynebacterium* species.^3^, ^4^

**Figure 1:**
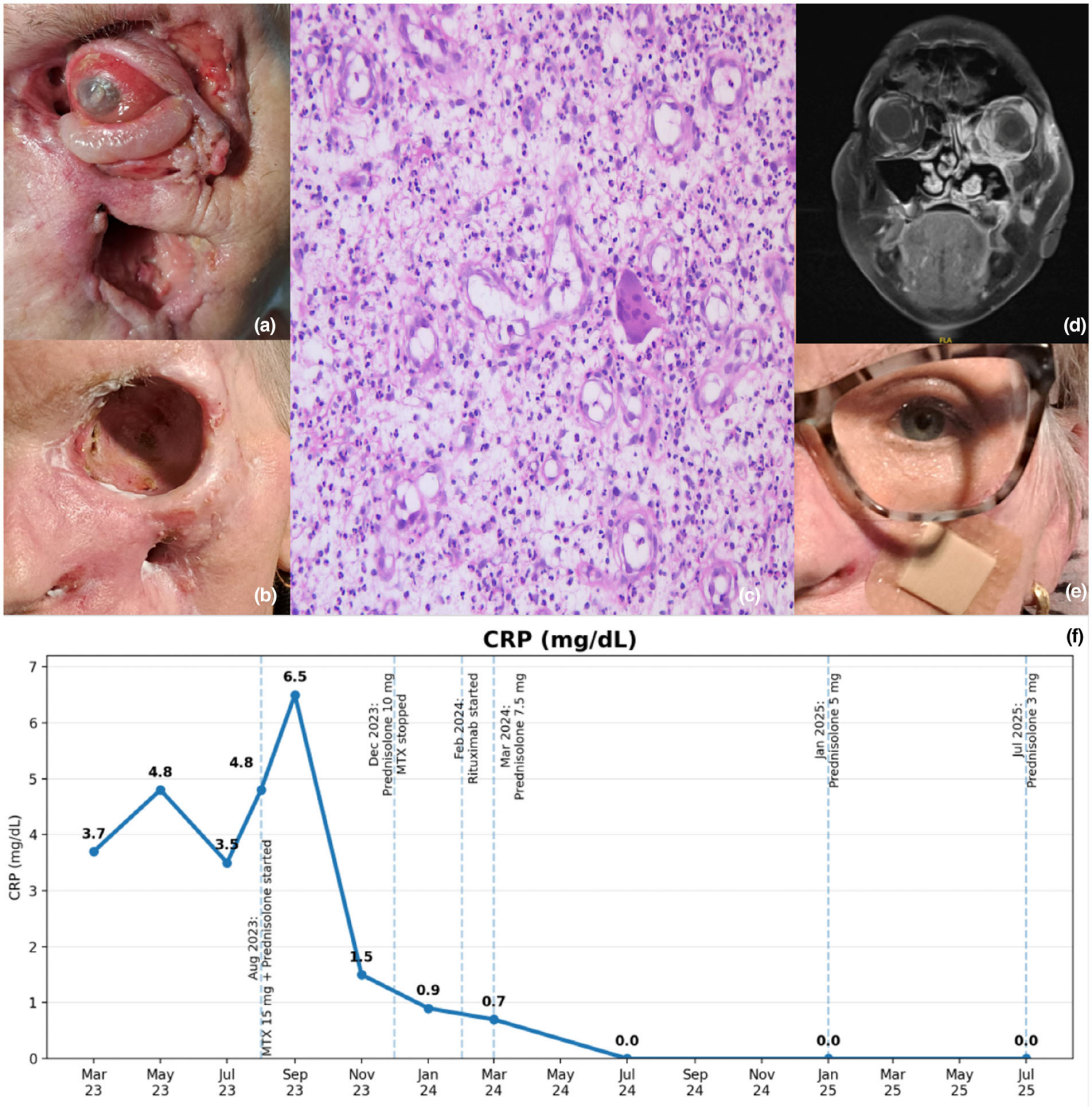
Clinical, radiological and histological disease course. **(a)** Baseline clinical presentation prior to the initiation of immunomodulatory therapy. **(b)** Clinical presentation 6 months post‐rituximab treatment and surgical defect reconstruction with a split‐thickness skin graft. **(c)** Histological examination of the maxillary sinus (20× magnification) demonstrating pronounced active chronic granulomatous and fibrosing inflammation with focal necrotizing features. **(d)** Magnetic resonance imaging (MRI) of the paranasal sinuses prior to immunosuppression, showing an extensive inflammatory‐destructive process in the left maxillary sinus with a large cutaneous fistula, ipsilateral cheek abscess formation, left periorbital phlegmonous inflammation and osseous destruction of the orbital floor and lamina papyracea. **(e)** Clinical presentation following aesthetic rehabilitation with an orbital epithesis. **(f)** Time course of serum C‐reactive protein (CRP) levels.

We report the case of a 79‐year‐old woman with a medical history of arterial hypertension and hypothyroidism who presented with rapidly progressive epiphora and purulent discharge from the left eye. Deep sterile tissue specimens obtained intraoperatively from the left cheek yielded *C. kroppenstedtii* on initial microbiological culture. The patient was subsequently treated with targeted antimicrobial therapy consisting of clindamycin for 2 weeks followed by vancomycin for 4 weeks. Despite subsequent microbiological evaluations confirming eradication of the organism, including follow‐up deep tissue sampling of the cheek and orbit performed 2 months after completion of antibiotic therapy, the patient's clinical condition continued to worsen. At that time, she was not receiving any systemic or local antibiotic treatment. Despite undergoing various escalating courses of treatment, including antibiotic treatment with levofloxacin 500 mg daily for 14 days and amoxicillin 1000 mg 3 times daily for 14 days, systemic antifungal treatment with fluconazole 400 mg daily for 6 weeks, local antifungal treatment with amphotericin B twice per day, followed by two local corticosteroid injections, the patient developed progressive phlegmonous inflammation of the left midface. Ultimately, intractable pain and severe functional impairment necessitated a left orbital exenteration (Figure 1a).

Computed tomography (CT), magnetic resonance imaging and positron emission tomography‐CT demonstrated extensive inflammatory destruction of the left maxillary sinus with cutaneous fistula and ipsilateral cheek abscess, causing significant functional impairment, particularly with eating (Figure 1d); no systemic or distant organ involvement was evident. The laboratory findings included moderately elevated inflammatory markers (Figure 1c) and absent eosinophilia. An extensive autoimmune workup was performed, including repeated testing for antineutrophil cytoplasmic antibodies (ANCA), rheumatoid factor (RF), anti‐cyclic citrullinated peptide antibodies (anti‐CCP antibodies) and cryoglobulins. All tests were negative, and ANCA remained repeatedly negative throughout the disease course. Multiple biopsies from the affected sinus and surrounding tissues showed florid erosive‐ulcerative, chronic granulomatous and fibrosing inflammation with multinucleated giant cells, focal necrosis and small‐vessel wall damage with inflammatory infiltration and focal fibrinoid necrosis, consistent with a necrotizing small‐vessel vasculitis (Figure 1c). No well‐formed granulomas were identified. Although histopathology confirmed vasculitis inflammation, differentiation between autoimmune and infectious vasculitis could not reliably be made on histomorphological grounds alone, as both entities exhibit overlapping inflammatory patterns.^5^

Five months after the initial detection of *C. kroppenstedtii*, repeated cultures from deep tissue specimens of the orbital region and cheek, fungal and mycobacterial diagnostics, blood and tissue cultures, special stains and molecular testing remained negative following the initial antibiotic treatment. The last two samples were collected without the influence of any systemic antibiotic or antifungal treatment. These findings made persistent infection‐associated vascular injury unlikely. The screening for malignancy and alternative midline destructive diseases, including upper and lower gastrointestinal endoscopy, revealed no significant findings.

Given the histopathological findings, progressive localized tissue destruction, negative autoantibody testing, and absence of ongoing infection, a diagnosis of ANCA‐negative localized necrotizing vasculitis was considered.

## Treatment

Systemic glucocorticoid therapy (prednisolone at 1 mg kg^−1^ day^−1^) led to marked clinical improvement and regression of local inflammation, supporting an immune‐mediated mechanism. Methotrexate (15 mg week^−1^) was added for steroid‐sparing but discontinued because of pancytopenia. Progressive disease prompted rituximab (1‐g IV infusions, 2 weeks apart), achieving partial remission, declining inflammatory markers and successful glucocorticoid taper; maintenance rituximab followed at regular intervals without infectious complications (Figure 1f).

After inflammatory stabilisation, exenteration of the non‐salvageable left orbit was performed because of irreversible structural destruction, followed by split‐thickness skin graft reconstruction (Figure 1b) and orbital prosthesis for aesthetic rehabilitation (Figure 1e). The patient remains in stable clinical remission on maintenance rituximab.

## Differential diagnosis

This case posed considerable diagnostic and therapeutic challenges because of negative autoantibody testing, an atypical strictly unilateral manifestation and lack of systemic involvement. Histology demonstrated features consistent with necrotizing small‐vessel vasculitis, and the marked response to glucocorticoids and rituximab further supports an immune‐mediated inflammatory process.

While a direct causal relationship remains unproven, the initial presence of *C. kroppenstedtii* may represent transient colonisation, a coincidental finding or an inflammatory trigger for the subsequent necrotizing vasculitis. Given the negative follow‐up cultures post‐treatment, persistent active infection is unlikely. Additionally, the possibility of an infection‐associated vascular injury cannot be excluded. Nevertheless, this observation highlights the importance of extended microbiological and molecular evaluation for fastidious lipophilic bacteria in destructive midline inflammatory lesions.

## Comment

To the best of our knowledge, this is the first report of destructive orbital disease presenting with ANCA‐negative necrotizing vasculitis and the initial detection of *C. kroppenstedtii*. While a direct causal link remains unproven, this case highlights the complexity of differentiating infection from immune‐mediated inflammation and underscores the necessity of exhaustive microbiological and immunological workups in atypical destructive inflammatory diseases.

## Author contributions

**Nadine Al‐Azem:** Writing – review and editing. **Inga Nierkamp:** Conceptualization. **Claudia Rudack:** Conceptualization. **Anne Kerlikowki:** Conceptualization. **Eva Wardelmann:** Conceptualization. **Martin A Kriegel:** Conceptualization. **Anna Knothe:** Conceptualization. **Rebecca Hasseli:** Writing – original draft; writing – review and editing; supervision; conceptualization. **Hermann Pavenstädt:** Conceptualization. **Jan‐Dirk Raguse:** Conceptualization. **Achim Georg Beule:** Conceptualization. **Friedericke Müller:** Conceptualization.

## Conflict of interest

The authors declare that they have no competing interests regarding this work.

## Informed consent

Written informed consent was obtained from the patient prior to taking photographs.

## Data Availability

Data sharing not applicable to this article as no datasets were generated or analysed during the current study.
